# Comparison of Efficacy of Platelet-Rich Plasma With and Without Topical Minoxidil for Hair Growth in Patients With Androgenetic Alopecia: A Prospective Study

**DOI:** 10.7759/cureus.53875

**Published:** 2024-02-08

**Authors:** Soorumsetty Ruthvik, Rubin S John, Melvin George, Santhosh P Kumar, Murugesan Krishnan

**Affiliations:** 1 Oral and Maxillofacial Surgery, Saveetha Dental College and Hospitals, Saveetha Institute of Medical and Technical Sciences, Saveetha University, Chennai, IND

**Keywords:** innovative practice, novel technique, androgenetic alopecia, hair growth, minoxidil, platelet rich plasma

## Abstract

Introduction

Androgenetic pattern of alopecia is a common problem occurring in men, which mostly arises from their younger age. There are many therapies advocated in the literature for hair loss reduction, and one of them is platelet-rich plasma (PRP) therapy. This study aimed to assess the efficacy of combined PRP therapy with topical minoxidil over PRP as monotherapy in hair loss reduction and regeneration of new hair.

Materials and methods

The study was conducted at our institute in the Department of Oral and Maxillofacial Surgery at Saveetha Dental College and Hospital. The study consisted of 40 participants, 20 of whom had only PRP therapy as part of their treatment, while the other 20 participants received PRP combined with topical minoxidil as treatment. Both group participants were evaluated for postoperative hair shaft diameter and hair follicle density. Parameters were measured preoperatively and postoperatively after one month, two months, and three months. Data analysis was done with the help of SPSS, with P-values less than 0.05 considered statistically significant. The Mann-Whitney U test was used to compare the two groups for measurement of hair shaft diameter, and for comparison between hair follicle density, an unpaired t-test was used.

Results

It was found that the mean hair shaft diameter in the PRP with minoxidil group was higher than that of the PRP group for one month (P = 0.023), two months (P = 0.001), and three months (P = 0.001) postoperative periods, and the results were statistically significant. Hair follicle density (mean hair quantity) was higher in the PRP group than in the PRP with the minoxidil group in the first postoperative month. However, this difference was not statistically significant (P = 0.08). While the mean hair quantity in the PRP with minoxidil group was higher than that in the PRP group for two months (P = 0.45) and three months (P = 0.001) postoperative periods, the results were statistically significant only at the three-month postoperative period.

Conclusion

It can be concluded that injectable autologous PRP with minoxidil as a topical agent is a better treatment option for the improvement of both hair quality (hair shaft diameter) and hair quantity (hair follicle density) compared to plain autologous injectable PRP monotherapy.

## Introduction

Androgenetic alopecia (AGA) is an increasing concern among men around the world. It is a disease characterized by progressive hair loss that is mediated by systemic androgens and genetic factors [1]. Factors like haphazard lifestyles and increased stress in both working and personal lives due to urbanization are considered aggravating factors for the early symptoms of AGA. Multiple options have been advocated for the treatment of hair loss, including low-level laser therapy, along with FDA-approved medications, minoxidil administered topically, and finasteride given orally [2]. Surgical techniques for the correction of AGA include hair follicular unit transplantation and hair follicular unit extraction techniques. Surgical techniques are expensive and technique sensitive, while with topical and oral medications, it takes time for the results to occur. This has led to dropouts from the treatment [3]. Hence, there is a need for minimally invasive and office-based procedures like autologous platelet-rich plasma (PRP).

PRP is basically a platelet concentrate consisting of growth factors obtained from autologous blood [4]. This PRP is injected into the scalp, especially in areas of hair loss, which will eventually stimulate hair regrowth. In vitro studies have demonstrated that after PRP therapy, there is an increase in the expression of beta-catenin, which is a key molecule in the signaling pathway that regulates hair follicle growth and hair matrix cell proliferation [5]. On the other hand, among patients who do not want to get any invasive procedure done on them, topical minoxidil is useful. Minoxidil is considered a treatment of choice in general for all patterns of hair loss, nonspecific to gender [6]. At a maximum concentration of 5%, minoxidil is available as an over-the-counter drug, making it more feasible for patients. Minoxidil is converted into an active metabolite form called minoxidil sulfate with the help of sulfotransferase enzymes present on the outer root sheath of the hair follicle [7]. The by-product formed (minoxidil sulfate) acts as a potent vasodilator and causes vasodilatation of the capillaries that supply the hair follicles, ultimately increasing their nourishment and promoting their growth [8,9]. It is essential to identify a better treatment modality for AGA, which includes monotherapy with topical minoxidil, autologous PRP, or a combination of both.

This study aimed to compare the efficacy of PRP therapy combined with topical minoxidil over PRP as monotherapy for patients with AGA in the reduction of hair loss and regeneration of new hair. The objectives of the study were to assess the hair shaft diameter and the hair follicle density in patients with AGA who were treated with PRP combined with topical minoxidil and PRP monotherapy.

## Materials and methods

Study design and setting

This prospective comparative study was conducted in the Department of Oral and Maxillofacial Surgery at Saveetha Dental College and Hospital. It was approved by the Institutional Human Ethical Committee, Saveetha Dental College (approval number IHEC/SDC/OMFS-2106/23/270), and informed consent was obtained from all the participants before the commencement of the study. According to the G*Power software, a total sample size of 40 was planned for the study. The total sample was divided into two groups, with 20 participants in each group. A simple random sampling technique was followed, and the allocation was done using an opaque envelope. PRP treatment for the participants in both groups was performed by the same surgeon, and the evaluation at periodic intervals was done by the same principal investigator for both groups.

Inclusion and exclusion criteria

AGA condition is categorized under the Hamilton Norwood classification from types 1 to 8 [2]. It is based on the recession patterns (frontoparietal, frontotemporal, and frontal recessions) of hair loss occurring in the scalp. All male participants with AGA (stages 1-4 of the Hamilton Norwood scale), aged 20-40 years, were included in the study. Patients who were categorized under stages 5-7 of the Hamilton Norwood scale were excluded from the study. Patients with autoimmune alopecia and systemic conditions like uncontrolled diabetes, hypertension, existing coagulopathies, anemia, malnutrition, and thyroid dysfunction were not enrolled in the study.

Procedure

**Figure 1:**
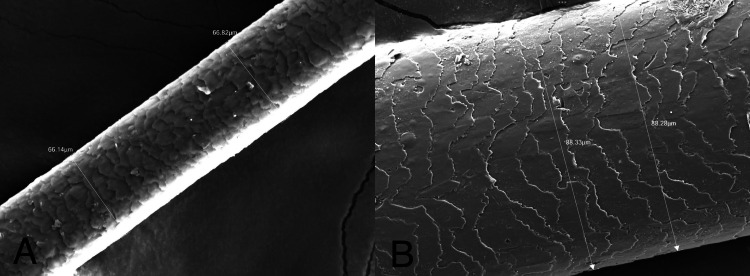
Scanning electron microscope views of hair shaft diameter. (A) Preoperative. (B) Third-month postoperative.

**Figure 2:**
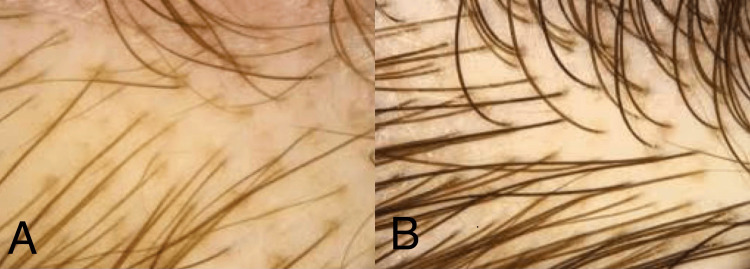
Trichoscope pictures showing hair follicle density. (A) Preoperative. (B) Third-month postoperative.

PRP was prepared from the autologous venous blood collected from the patient. A total of 20 ml of blood was collected in two tubes coated with anticoagulant. The tubes were immediately centrifuged at a speed of 1,000 rpm for 10 minutes. Slow speed was chosen in the beginning to prevent the displacement of platelets to the bottom layer. The plasma was collected in a single tube and again centrifuged at 3,500 rpm for 10 minutes. The principle behind the centrifugation was to separate the collected blood into three layers: RBCs at the bottom, acellular plasma (platelet-poor plasma) at the top, and a platelet-rich buffy coat layer in the middle. The plasma near and above the central buffy coat was collected into insulin syringes. Preoperatively, the following stable landmarks were marked: the glabella (soft tissue midpoint between the eyebrows), the inion (highest point on the occipital protuberance), and the upper tragal points. The line joining from the glabella to the inion and the line extending from both the tragal points intersect at a point, forming a junction. This area was considered the study area for future sample collection and trichometer analysis. PRP collected in the insulin syringes was injected into the areas of hair loss. Small amounts of PRP were injected into the subcutaneous layer of the scalp. Although local anesthesia can be administered, it was not preferred, and alternatively, ice packs were used for pain relief and vasoconstriction. For Group 1, no topical agent was prescribed, and PRP was injected every month periodically, while for Group 2, along with periodic PRP treatment, topical minoxidil was prescribed for daily usage. Treatment in both groups was carried out for up to three months. The two parameters that were assessed in the follow-up period were hair follicle density and hair shaft diameter. Hair shaft diameter was measured with the help of a scanning electron microscope (JEOL FE-SEM Version 2, JEOL, Ltd., Tokyo, Japan), as shown in Figure 1. Hair follicle density was assessed with the help of a cross-section trichometer, as depicted in Figure 2. Parameters were measured preoperatively and at the first, second, and third postoperative months.

Statistical analysis

Data analysis was done with the help of IBM SPSS Statistics for Windows, Version 22.0 (Released 2013; IBM Corp., Armonk, NY, USA), with P-values less than 0.05 considered statistically significant. The Mann-Whitney U test was used to compare between two groups for measurement of hair shaft diameter, and for comparison between hair follicle density between the groups, an unpaired t-test was used.

## Results

**Figure 3:**
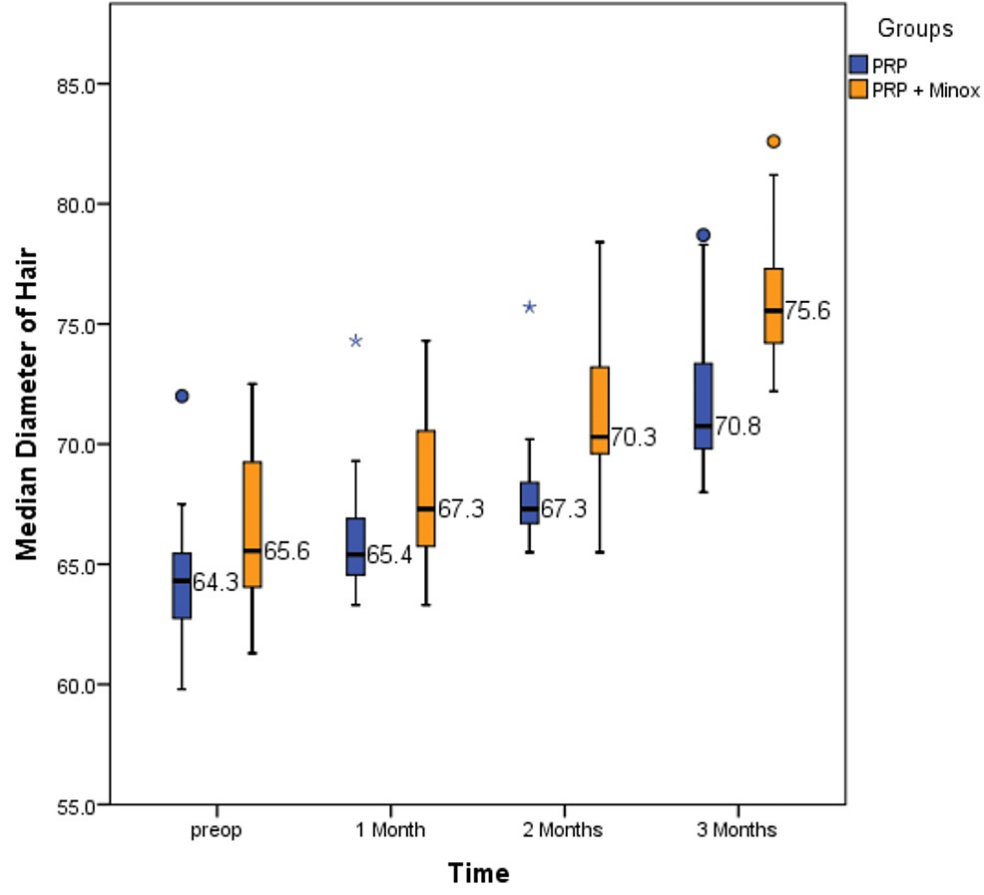
Comparison of the median diameter of the hair shaft using the scanning electron microscope between the PRP group and the PRP with minoxidil group PRP, platelet-rich plasma; PRP + minox, PRP with minoxidil

Our study consisted of 40 participants with a mean age of 35 ± 5.5 years. The present study had two groups: Group 1 (N = 20), in which plain autologous PRP was given without any topical agents, and Group 2 (N = 20), in which, along with PRP therapy, topical minoxidil was given on a daily basis. Postoperatively, hair follicle density and hair shaft diameter were measured at regular follow-up intervals. Preoperatively, there was no statistically significant difference in mean hair shaft diameter between the PRP group and the PRP with minoxidil group (P = 0.056). It was found that the mean hair shaft diameter in the PRP with minoxidil group was higher than that of the PRP group for one month (P = 0.023), two months (P = 0.001), and three months (P = 0.001) postoperative periods, respectively, and the results were statistically significant. Table 1 and Figure 3 represent the results obtained among the two groups with respect to hair shaft diameter.

**Table 1:** Comparison of the median diameter of the hair shaft using a scanning electron microscope between the PRP group and the PRP with minoxidil group Statistically significant at *P < 0.05 using the Mann-Whitney U test IQR, interquartile range; NS, not significant; PRP, platelet-rich plasma; PRP + minoxidil, PRP with minoxidil

Timelines	Groups	N	Median	IQR	Mann-Whitney U statistic	P value
Preoperative	PRP	20	64.3	62.5-65.4	129.5	P = 0.056
	PRP + minoxidil	20	65.5	63.8-69.6		NS
One month postoperative	PRP	20	65.45	64.4-67.1	116.5	P = 0.023*
	PRP + minoxidil	20	67.3	65.35-70.7		
Two months postoperative	PRP	20	67.3	66.7-69.1	39.5	P = 0.001*
	PRP + minoxidil	20	70.75	69.7-74		
Three months postoperative	PRP	20	70.75	69.7-73.4	61.5	P = 0.001*
	PRP + minoxidil	20	75.55	74.2-77.7		

**Figure 4:**
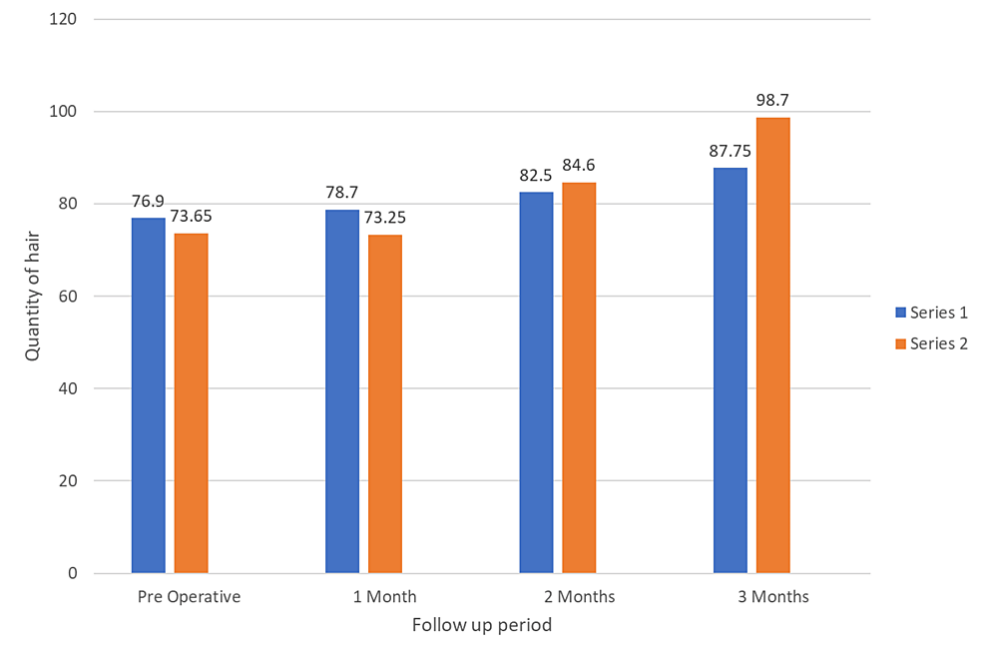
Comparison of the mean quantity of hair between the PRP group and the PRP with minoxidil group as assessed using a trichometer PRP, platelet-rich plasma; Series 1, platelet-rich plasma group; Series 2, platelet-rich plasma with minoxidil group

Preoperatively (P = 0.31) and for the one-month postoperative period (P = 0.08), the mean hair quantity for the PRP group was higher than that of the PRP with minoxidil group. However, this difference was not statistically significant. After the two-month postoperative period (P = 0.45), the mean hair quantity for PRP with the minoxidil group was higher than that of the PRP group, and this difference was not statistically significant. However, during the three-month postoperative period, the mean hair quantity for PRP with the minoxidil group was higher than the mean hair quantity for the PRP group, and this difference was statistically significant (P = 0.001). Table 2 and Figure 4 represent the results obtained among the two groups with respect to the mean quantity of hair.

**Table 2:** Comparison of the mean quantity of hair between the PRP group and the PRP with minoxidil group as assessed using a trichometer Statistically significant at *P < 0.05 using an unpaired t-test NS, not significant; PRP, platelet-rich plasma; PRP + minoxidil, PRP with minoxidil; SD, standard deviation

Timelines	Groups	Number	Mean	SD	t	P value
Preoperative	PRP	20	76.9	10.5	1.018	P = 0.31
	PRP + minoxidil	20	73.65	9.89		NS
One month postoperative	PRP	20	78.7	10.3	1.77	P = 0.08
	PRP + minoxidil	20	73.25	8.9		NS
Two months postoperative	PRP	20	82.5	10.02	-0.76	P = 0.45
	PRP + minoxidil	20	84.6	7.1		NS
Three months postoperative	PRP	20	87.75	9.03	-3.67	P = 0.001*
	PRP + minoxidil	20	98.7	9.7		

## Discussion

PRP therapy is a very cost-effective and simple chairside treatment for the management of hair loss. PRP injections for the treatment of patients with AGA are provided with platelet-rich concentrate [10]. There are several biomolecules and growth factors that are produced either from the activation of platelets, the plasma, or both combined. Activated platelets have microparticles, growth and mitogenic factors, cytokines, chemokines, and related compounds, adhesive proteins, proteases, and antiproteases. They are proven to have a combined effect on hair growth and also prevent hair fall [7]. In a systematic review, 21 out of 24 studies used PRP to assess its potential for hair restoration, and all the studies showed positive results with PRP for hair follicle density and hair quality improvement, as the results were statistically significant [11]. In the course of our study, it was shown that all the patients undergoing PRP therapy received good results with respect to hair follicle density and hair shaft diameter, but PRP with minoxidil showed superior results over autologous PRP with respect to hair shaft diameter and hair follicle density.

In our study, the combined use of PRP with minoxidil is of particular interest. In a similar study, it was proven that the use of PRP in combination with other standard treatment options like minoxidil or finasteride gave pronounced results, which were confirmed by trichoscopy alone [12]. Most of the published literature has used a similar measuring strategy [13-15], whereas in our study, the latest version of the scanning electron microscope was used to evaluate the hair shaft diameter, which is a novel approach. Not only the significant improvement in the hair density but also the individual hair shaft diameter increment was also noted in the groups. Assessment of individual hair shaft health at the site of intervention with PRP and PRP with minoxidil was not compared before in any available literature. The evaluation of individual hair shaft diameter is an indirect indicator for the assessment of hair follicle health. The hair shafts were collected from the same site at every follow-up visit, and the lower third of the hair shaft diameter was measured using the latest generation of scanning electron microscopes.

Minoxidil does not directly act on the levels of testosterone or adrenal androgen secretion and does not alter the genetically programmed sensitivity of hair follicles toward the androgens [16]. Sulfotransferases are the enzymes of the scalp that convert minoxidil into its active metabolite, which will stimulate hair growth. Minoxidil will cause vasodilation and provide more oxygen and nutrients to the hair follicles, leading to healthy hair growth. The combination of PRP and minoxidil has a synergistic effect on hair follicle density and hair shaft diameter [17].

Limitations of the study

A limitation of our study will be a short follow-up period. Future studies with a longer follow-up period to assess the efficacy of PRP and minoxidil in the maintenance phase are recommended.

## Conclusions

It can be concluded from the study that injectable autologous PRP with minoxidil as a topical agent is a better treatment option for increasing both hair quality (hair shaft diameter) and hair quantity (hair follicle density) than plain autologous injectable PRP. Further studies with a larger sample size and a longer follow-up period are necessary to obtain more accurate results and formulate a better treatment protocol for the management of AGA.
